# Quality Assessment during Incubation Using Image Processing

**DOI:** 10.3390/s20205951

**Published:** 2020-10-21

**Authors:** Sheng-Yu Tsai, Cheng-Han Li, Chien-Chung Jeng, Ching-Wei Cheng

**Affiliations:** 1 Department of Bio-Industrial Mechatronics Engineering, National Chung Hsing University, Taichung 402, Taiwan; d105040001@mail.nchu.edu.tw (S.-Y.T.); d108040001@mail.nchu.edu.tw (C.-H.L.); 2 Department of Physics, National Chung Hsing University, Taichung 402, Taiwan; ccjeng@phys.nchu.edu.tw; 3 College of Intelligence, National Taichung University of Science and Technology, Taichung 404, Taiwan

**Keywords:** incubation, spectrum, image processing, ROC curve

## Abstract

The fertilized egg is an indispensable production platform for making egg-based vaccines. This study was divided into two parts. In the first part, image processing was employed to analyze the absorption spectrum of fertilized eggs; the results show that the 580-nm band had the most significant change. In the second part, a 590-nm-wavelength LED was selected as the light source for the developed detection device. Using this device, sample images (in RGB color space) of the eggs were obtained every day during the experiment. After calculating the grayscale value of the red layer, the receiver operating characteristic curve was used to analyze the daily data to obtain the area under the curve. Subsequently, the best daily grayscale value for classifying unfertilized eggs and dead-in-shell eggs was obtained. Finally, an industrial prototype of the device designed and fabricated in this study was operated and verified. The results show that the accuracy for detecting unfertilized eggs was up to 98% on the seventh day, with the sensitivity and Youden’s index being 82% and 0.813, respectively. On the ninth day, both accuracy and sensitivity reached 100%, and Youden’s index reached a value of 1, showing good classification ability. Considering the industrial operating conditions, this method was demonstrated to be commercially applicable because, when used to detect unfertilized eggs and dead-in-shell eggs on the ninth day, it could achieve accuracy and sensitivity of 100% at the speed of five eggs per second.

## 1. Introduction

Eggs—rich in protein, minerals, and vitamins, and an important source of nutrients for humans—play a vital role in the human daily diet. The importance of fertilized eggs is increasing because fertilized eggs are currently used to manufacture most vaccines, wherein fertilized eggs are usually screened and delivered to vaccine manufacturers by the 10th day [1,2,3]. A fertilized egg takes about 21 days to hatch, with the chick embryo completely formed after the 18th day. Fertilized eggs are generally screened via light illumination. With the light penetrating the shell, the embryo development is judged. The eggs are preliminarily classified as fertilized and unfertilized; the fertilized eggs can be further divided into normal and dead-in-shell embryos. Artificial screening mainly aims to remove unfertilized and dead-in-shell eggs. Eggs are usually subjected to three light-based inspections during the hatching process. The first inspection is conducted on the sixth day, when spiderweb-like blood vessels develop along the eggshell membrane inside the normally fertilized eggs. In contrast, the interior of an unfertilized egg is devoid of blood vessels and appears bright when held against light, while only a black blood clot-like partial embryo can be identified in a dead-in-shell egg. The second inspection is conducted on the 10th day; dead-in-shell eggs with only tiny blood vessels, if found, are removed to avoid the malodorous amine gas produced by the fermentation of eggs due to long-term decomposition of dead embryos, which can cause the eggs to burst and generate pollution. To eliminate embryos that might die after the 10th day, the third inspection is conducted on the 18th day, when the eggs are about to be placed in the hatching room; subsequently, these eggs are removed to avoid resource wastage. Moreover, non-removal of dead embryos can pollute the hatching environment and reduce the hatching rate [4].

Recently, there have been numerous nondestructive-detection-based studies on monitoring the hatching process of eggs, including machine vision, percussion-vibration methods [5], optical detection method, and dielectric-characteristic measurement method. To develop a nondestructive detection technique capable of distinguishing fertilized eggs from eggs not suitable for vaccine production, LEDs can be used where visible light and near-infrared (VNIR) spectroscopy techniques are employed in conjunction with a LED light source [3]. In [6], the authors used machine vision to identify whether an egg was fertilized. In their study, a LED lamp was used to penetrate the eggshell at a close range, with a CCD camera used to film the embryo growth inside the egg. Using an image-processing method, relevant characteristics, such as the growth of blood vessels inside the eggs, were obtained as the basis for identification. The identification accuracy rates corresponding to the 1st–5th days of incubation were 47.13%, 81.41%, 93.08%, 97.73%, and 98.25%, respectively. The optical detection method has a wide range of applications, including the detection of the sugar content, pH value, and water content [7,8,9] of agricultural products; VNIR spectroscopes are usually employed as a nondestructive light source to detect the protein quality and freshness [10,11] of egg products. Whether an egg is fertilized [12,13] is determined by the light absorption ratios of different egg compositions, such as eggshell, egg white, blood vessels, and erythrocytes developed during the embryo growth inside the egg as they exhibit different absorption spectral bands. In the optical detection method, similar to machine vision, one side of the egg is illuminated with strong light, and a CCD camera placed on the other side to detect the absorption spectral wavelength of the egg to determine the fertilization and development status. Using a 50-W tungsten halogen lamp to illuminate the eggshell, Liu and Ngadi collected spectral images in the wavelength range of 900–1700 nm using a NIR hyperspectral imaging system, with the signals filtered to determine the egg status [4]. Using a similar method to collect data on spectral wavelengths between 400 and 1000 nm for identification, Smith et al. achieved an identification accuracy rate of 71%, 63%, 65%, and 83% on the 0th–3rd day of incubation, respectively [14]. Collecting data on wavelengths between 400 and 1000 nm, Zhu et al. extracted 155 spectral characteristic variables from the 520-nm waveband and used a support vector machine (SVM) to classify and model the image, spectrum, and image-spectrum fusion information to identify fertilized eggs, unfertilized eggs, and dead embryos, with the identification accuracy rate being 84%, 90%, and 93%, respectively. They concluded that the image-spectrum fusion information-based accuracy was higher than the single characteristic identification-based accuracy [15]. In the dielectric-characteristic measurement method, a high-frequency wave was input through a parallel plate without destroying the eggshell, where characteristic values, such as the dielectric constant and loss factor of the egg at different frequencies, were continuously measured and imported into an artificial neural network and an SVM classifier to classify the samples [16].

In this study, an image-processing method was used to analyze egg spectra. In this method, a 590-nm-wavelength LED was selected as the light source to obtain sample data, where sample colors were layered and converted into grayscale images using an imaging device, and a receiver operating characteristic (ROC) curve was employed to analyze the daily data to obtain the area under the curve (AUC) as the basis for determining the optimal screening threshold. Finally, the actual operation and verification were conducted using the detection device developed in this study.

## 2. Materials and Methods

### 2.1. Sample and Experimental Equipment

The eggs used in the experiment were of the Lohmann variety and were cold-stored and had not started hatching (0-day age). They were randomly selected at the moment of purchase. Two main experiments were conducted in this study. The first experiment aimed to seek the most suitable light source for image processing, with ten fertilized eggs and four unfertilized eggs used. In the second experiment, 150 eggs were used to establish the threshold, with the other 150 eggs used for verification. The environmental parameters of the incubation equipment were set in accordance with the literature, with temperature and humidity set to 100 °C and 70% [5,17], respectively.

**Figure 1:**
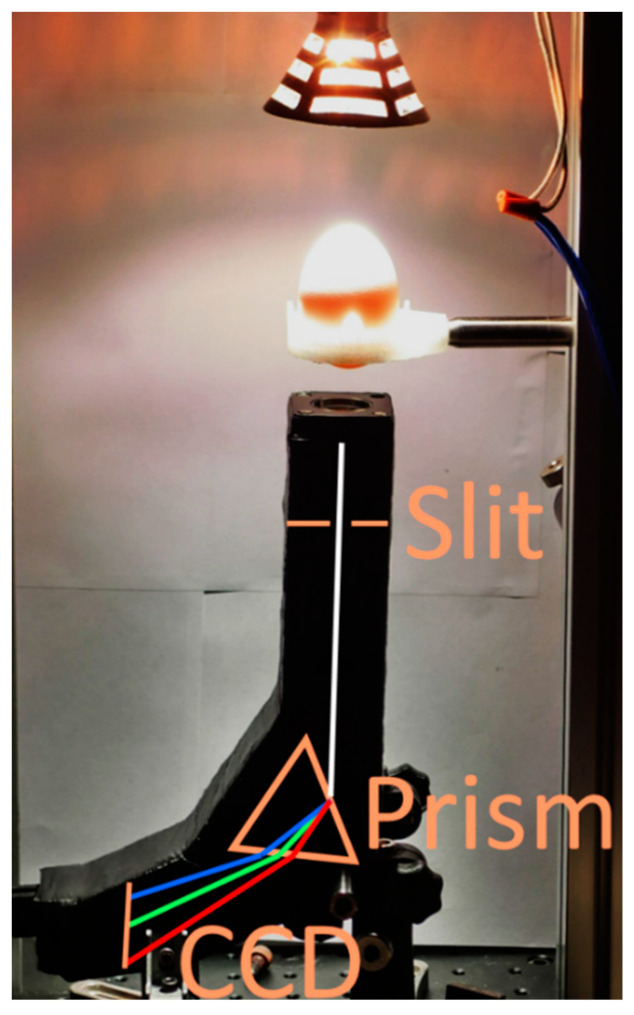
Spectrum experimental setup.

In Experiment 1, a 50-W halogen lamp with an illuminance of 2300 lux was used as the light source. After passing through the egg, the light passed through an equilateral dispersive prism and was received by a CCD imaging sensor (ICX274, Sony, size type: 1/1.8, 1600 × 1200 pixels). The spectral wavelength of the received sample is shown in Figure 1. In [18], the authors used a spectrometer and a 475–810-nm mercury neon lamp to calibrate the wavelength. This setup was used to determine the freshness of brown-shelled and white-shelled eggs. After obtaining the light wavelength penetration response spectra of brown-shelled and white-shelled eggs, the spectral data were processed using standard normal variate (SNV). Finally, multiple regression analysis (MLR) was used to classify and judge the freshness of white and red eggs. Experiment 2 of this study aimed to develop the detection device. According to the results of the Experiment 1, a 5-W LED lamp with a wavelength of 590 nm was used as the light source. The images were taken with a color camera manufactured by ACTi Corporation (model no. E23) with a resolution of 1920 × 1080 (2 million pixels). This development device has 50 LEDs inside. When the whole plate of eggs (150 eggs) passes, the camera at the bottom takes pictures, analyzes the data in real time, and displays the identification results of the whole plate.

### 2.2. Experimental Apparatus and Measuring Methods

#### 2.2.1. Experiment 1

In this experiment, ten fertilized eggs and four unfertilized eggs were hatched simultaneously, with the spectra of the eggs on the 1st–9th day of incubation measured to analyze the daily trend. With wavelengths between 320 and 1100 nm, a halogen lamp was used in this experiment to seek the absorption spectra of the fertilized eggs. The captured images were analyzed using the software developed by Andor Technology Ltd. to understand the changing trend of the absorption spectra. This experiment mainly aimed to identify a low-watt lamp with a single wavelength most suitable for the eggs as the light source of the detection device. Low-power LEDs were used as high-power LEDs can cause heat dissipation and the light passes through the egg with too much power, seriously overexposing and affecting the surrounding eggs; this causes serious problems in sampling the entire screen. The industrial prototype of the device designed and fabricated in this study would require 50 LEDs.

#### 2.2.2. Experiment 2

**Figure 2:**
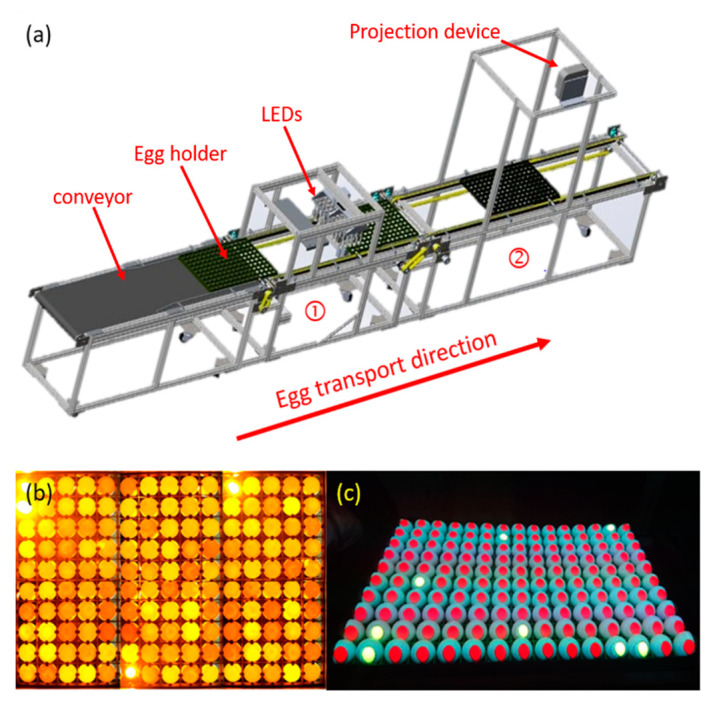
(**a**) Detection device (**1**) the image-capturing zone; and (**2**) the projection marking zone); (**b**) schematic of image capturing; and (**c**) schematic of projection marking.

In this experiment, 150 unselected eggs were incubated simultaneously, with the eggs filmed daily from the inception of incubation. Following the hatching, the unfertilized, fertilized, and dead-in-shell eggs were classified, and daily ROC curves were established to determine the screening threshold value. Finally, the accuracy of the detection equipment was verified using the other 150 unselected eggs. Figure 2a shows a three-dimensional view of the detection device drawn using SolidWorks. The main function of Section 1 of the device is to capture images and detect the 150 eggs, as exhibited in Figure 2b. Following the detection, the eggs are transferred to Section 2 through the conveyor belt, where the colors are projected on eggs by the projection device, as shown in Figure 2c, with unqualified eggs removed manually.

#### 2.2.3. Digital Image Processing

A digital image comprises array pixels, and image position pixels can be represented by a matrix. A pixel comprises three primary colors: Red (R), Green (G), and Blue (B). The colored image can be converted to a grayscale image by converting the RGB color space into the YIQ color counterpart, where Y indicates luminance, representing the brightness of the light; I indicates the channel of in-phase; and Q indicates the quadrature phase, representing the color details. The color image can be converted into the brightness of the three primary colors, individually, using the formula  Y(m,n)=0.299·R(m,n)+0.587·G(m,n)+0.114·B(m,n), with the brightness ranging from 0 to 255, where 0 represents full darkness, 255 indicates full brightness, and the brightness value is the grayscale value [19]. In this study, images of the bottom of the eggs were taken, and each egg was positioned separately. The individual values could be obtained from the grayscale value from the red layer; these individual values were used as the basis for the selection of fertilized, unfertilized, and dead-in-shell eggs.

#### 2.2.4. Statistical Analysis

The ROC curve (statistical analysis) is a receiver operating characteristic curve [20] or a relative operating characteristic curve. It is a coordinate graphical analysis tool used for selecting the best signal detection model as well as setting the optimal threshold value in the same model. Without being influenced by costs or effectiveness, the ROC analysis gives objective and neutral advice [21] to help users make a decision. In 1997, Hanson indicated that the AUC [22,23] could be used to describe the accuracy of the risk assessment scale. Therefore, in this study, the RGB-layered images of egg samples (numbered) of each day were used to calculate the average grayscale value of each egg’s red layer. After the sample eggs were hatched, the unhatched eggs were sorted. The samples were divided into fertilized, unfertilized, and dead-in-shell eggs, and ROC classification was performed with the values of unfertilized and fertilized eggs to obtain the AUC value of unfertilized eggs each day. Next, the values of dead-in-shell and fertilized eggs were used. After ROC classification, the AUC value on each day for dead-in-shell eggs was obtained. Finally, the number of days for which AUC is maximum was determined and used to evaluate the accuracy of the classification results.

The ROC curve analyzes a binary classification model, that is, a model with only two types of output results. To evaluate the accuracy and sensitivity of the detection device, the detection device-based and actual classification results were divided into four categories using a confusion matrix, as shown in Table 1. The unfertilized eggs were taken as an example for explanation. The unfertilized eggs confirmed by the detection device were found to be unfertilized; in this setting, unfertilized eggs are considered as True Positive (TP). The unfertilized eggs detected to be not unfertilized proved to be not unfertilized; in this setting, unfertilized eggs are considered as True Negative (TN). The unfertilized eggs detected to be unfertilized proved to be not unfertilized; in this setting, unfertilized eggs are considered as False Positive (FP). The unfertilized eggs detected to be not unfertilized were found to be unfertilized; in this setting, unfertilized eggs are considered as False Negative (FN) [23].

The optimal discrimination threshold is an important indicator used to evaluate the ROC curve. The ROC curve is drawn using “sensitivity” as the y-axis and “1 − specificity” as the x-axis [18]. The AUC has values ranging from 0 to 1. A larger AUC value indicates a higher accuracy. In real life, the random-guess AUC value for a dichotomy problem is not less than 0.5 [23]. In this study, ROC curves were used to determine the maximum AUC, i.e., the judgment threshold value.

Youden’s index uses the comprehensive performance of sensitivity and specificity to determine the optimal discrimination threshold and calculates the value of “Sensitivity + Specificity − 1”. The calculated values range from 0 to 1; the closer the value is to 1, the better is the overall performance of sensitivity and specificity, where Sensitivity=TP/(TP+FN) is defined as the probability of a correct prediction in a group with true results and Specificity=TN/(FP+TN) is defined as the probability of correct prediction in groups with false results [24,25].

## 3. Results and Discussion

### 3.1. Experiment 1

**Figure 3:**
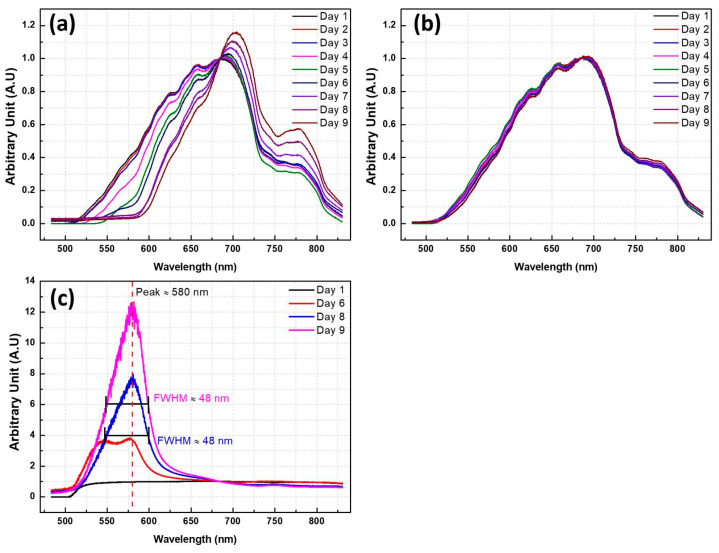
(**a**) Average values of fertilized egg spectral data on Days 1–9; (**b**) average values of unfertilized egg spectral data on Days 1–9; and (**c**) average values of unfertilized eggs divided by average values of fertilized eggs.

This experiment investigated the spectra of 14 eggs—ten eggs fertilized and the other four unfertilized. A halogen lamp was used to illuminate the incubated samples, and an image-capturing device was employed to record the data on Days 1–9. Then, Andor software was employed to analyze and obtain the data on spectral change on a daily basis, as shown in Figure 3. The average daily change trend of fertilized eggs is shown in Figure 3a, clearly indicating that, in the wavelength range below 680 nm, the values decrease as the number of incubation days increases, while Figure 3b exhibits the average daily change trend of unfertilized eggs, suggesting that the values show no significant change in the absorption spectra as the number of incubation days increases. Therefore, the values of unfertilized eggs, regarded as control samples, were compared with those of the fertilized eggs, as shown in Figure 3c. Dividing the grayscale values of the daily mean value of unfertilized eggs by the grayscale values of the daily mean value of fertilized eggs indicates that the peak of the ratio of absorption spectra of fertilized eggs is close to 580 nm. This band falls in the wavelength range of orange to yellow light; accordingly, a 590-nm-wavelength LED lamp was used in the detection device as it has a wavelength closer to 580 nm.

### 3.2. Experiment 2

**Figure 4:**
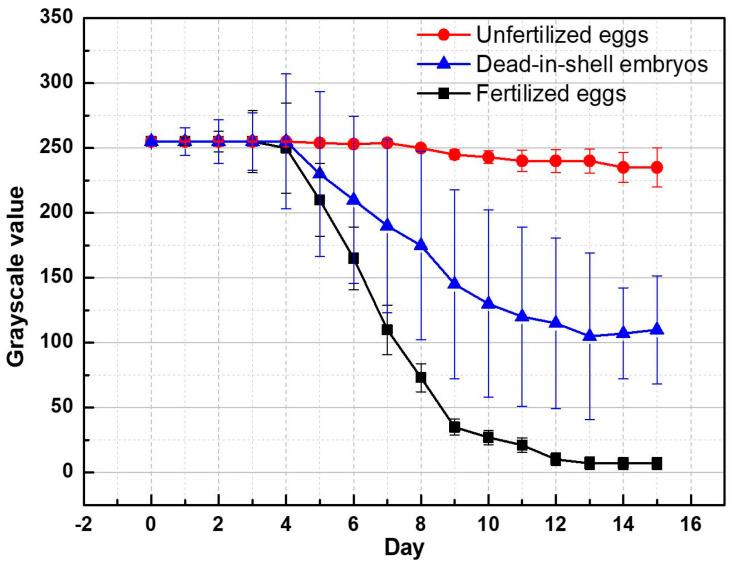
Change in grayscale values of fertilized eggs, unfertilized eggs, and dead-in-shell eggs.

In this experiment, the classification basis for the detection device was established using 150 eggs—18 unfertilized eggs, 12 early dead-in-shell eggs, and 120 successfully hatched eggs. The red layer in the images of the sample eggs on the 1st–15th days of incubation, captured using the detection device, were converted into grayscale counterparts through image processing. The change in grayscale values was recorded daily, with the average values shown in Figure 4, which indicates that the grayscale values decrease during the incubation process due to the continuous growth of embryos. Finally, the ROC curves were used to analyze the individual binary classification of unfertilized and dead-in-shell eggs from normally fertilized eggs, as shown in Table 2. The grayscale values of the unfertilized and dead-in-shell eggs can be clearly distinguished from those of the normally fertilized eggs from Day 7 onward. The AUC value of both reached 0.99 on Day 9, indicating that the method has an outstanding discrimination capability. In 2015, Kimura et al. used LED lamps with 585- and 635-nm wavelengths as the light sources to screen eggs on the 12th day of incubation. In their experiment, the detection accuracy rate was 92.9% under a single light source and 75% [3] when 36 light sources were used. In contrast, unfertilized eggs and dead-in-shell eggs could be identified with an overall accuracy rate of 100% on the ninth day of incubation, when using the light source developed in this study.

With the ROC analysis results imported into the detection device, the accuracy was verified using 150 eggs. The numbers of unfertilized and dead-in-shell eggs determined by the detection device were individually and statistically analyzed. In this experiment, 123 eggs were successfully hatched, of which 11 were unfertilized, 7 were early dead-in-shell, and the remaining 9 were confirmed by dissection to be late dead-in-shell (the chicks failed to move out of the shell). This study aimed to detect unfertilized and dead-in-shell eggs at an early stage. The late-stage dead-in-shell eggs fell in the category of normal eggs, as there were nearly mature chick embryos in the shells; that is, the embryos developed normally during the first half of the incubation period and the chicks failed to break shells just due to individual factors. According to the results in Figure 4, it is obvious that a threshold (grayscale value) can be used to classify unfertilized eggs from fertilized and dead-in-shell eggs (150 in total). Table 3 shows the results of the identification and classification of unfertilized eggs. After removing 11 unfertilized eggs, another threshold can be used to classify dead-in-shell eggs from fertilized eggs (139 in total). Table 4 shows the results of the identification and classification of dead-in-shell eggs.

In Figure 4, the grayscale values of the fertilized eggs began to drop on the fourth day, increasing the difference in the values between fertilized and unfertilized eggs. Table 3 also indicates that the accuracy of detecting unfertilized eggs on the seventh day was up to 98%, with a sensitivity of 82% and a Youden’s index of 0.813, and that both the accuracy and sensitivity reached 100%, with Youden’s index reaching 1 on the ninth day, suggesting that the judgment accuracy increases day-by-day. Table 4 exhibits the results of detecting dead-in-shell eggs. Note that the sensitivity reached the peak value on Day 9. At this moment, both accuracy and sensitivity reached 100%, with Youden’s index reaching 1. The embryos in dead-in-shell eggs stopped growing due to sudden death. However, the difference in grayscale values between dead-in-shell eggs and normally fertilized eggs on the 5th–7th days was not significant. Generally, the difference was not significant until Day 8, when the accuracy, sensitivity, and Youden’s index reached 97.8%, 71.4%, and 0.693, respectively. The detection result was worse than that on the ninth day, which was mainly accounted for by the fact that each egg developed at its own speed, causing the numbers and formation rates of blood vessels and hemoglobin molecules to be different. On the 10th day, the sensitivity dropped to 85.7%, which was mainly due to the misjudgment caused by the erythrocyte protein deterioration resulting from dead embryos in the dead-in-shell eggs [12,13], which changed the absorption spectrum of the substance. Therefore, according to this study’s results, the two grayscale values on the ninth day can be used as the threshold to effectively classify unfertilized and dead-in-shell eggs.

## 4. Conclusions

In this study, a spectral wavelength of 580 nm proved to be favorable for the detection of egg embryos, inspiring us to use a 5-W LED lamp with a wavelength of 590 nm as the detection light source, as the LED lamp has a wavelength closer to 580 nm. Using this device, we extracted data on 150 eggs, with ROC curves and AUC values used as a reference to obtain the daily optimal discrimination threshold values suitable for detecting unfertilized and dead-in-shell eggs. The detection device developed in this study was verified using another 150 eggs and was found to be capable of identifying the unfertilized eggs on the seventh day with an accuracy of 98% and a sensitivity of 82% at a screening threshold of 190.5, while dead-in-shell eggs could be identified on the ninth day with an accuracy of 100% and a sensitivity of 100% at a screening threshold of 74. At present, most vaccines are made from eggs, which are generally screened and delivered by the hatchery personnel to vaccine suppliers by the 10th day. Therefore, if 182.5 and 74 are used as the screening thresholds, unfertilized and dead-in-shell eggs, which are not suitable for vaccine culture, could be removed on the ninth day. With the developed screening thresholds introduced into the detection device, 150 eggs could be detected at a time—the eggs were detected at a speed of 30 s per tray, or five eggs per second, at an accuracy of 100%. The detected eggs were then marked with projected colors, which enabled the users to effectively screen eggs. This research provides a fast and accurate detection method. If commercial detection equipment is developed in the future, the target sample can be effectively detected using the research results’ screening threshold.

## 5. Patents

We successfully obtained the Taiwanese invention patent “Method for judging the hatching shape of poultry eggs by using image processing”, patent number TWI644616B.

## Figures and Tables

**Table 1:** Prediction and allocation table and related formulas.

			Real State	
			True	False
**Predict**		**True**	*True Positive (TP)*	*False Positive (FP)*
		**False**	*False Negative (FN)*	*True Negative (TN)*
$Accuracy=\frac{TP+TN}{TP+TN+FP+FN}$	$Precision=\frac{TP}{TP+FP}$	$Sensitivity=\frac{TP}{TP+FN}$	$Specificity=\frac{TN}{TN+FP}$	$Negative\;Prediction=\frac{TN}{TN+FN}$

**Table 2:** ROC analysis results.

	ROC	Day 7	Day 8	Day 9	Day 10
**Unfertilized eggs**	Grayscale values	190.5	188.0	182.5	181.0
	AUC	0.97	0.99	0.99	0.99
**Dead-in-shell embryos**	Grayscale values	122.0	107.5	74.0	65.0
	AUC	0.83	0.95	0.99	0.99

**Table 3:** Using detection device to identify and classify unfertilized eggs on the 7th–10th day.

Day 7		**Real State**		Grayscale Values: 190.5		AUC: 0.97
		**TRUE**	**FALSE**	**Total**	**Accuracy (%)**	**Precision (%)**
**Predict**	**TRUE**	9	1	10	98.0	90.0
	**FALSE**	2	138	140	**Sensitivity (%)**	**Specificity (%)**
**Total**		11	139	150	81.8	99.3
Day 8		**Real State**		Grayscale Values: 188.0		AUC: 0.99
		**TRUE**	**FALSE**	**Total**	**Accuracy (%)**	**Precision (%)**
**Predict**	**TRUE**	10	0	10	99.3	100.0
	**FALSE**	1	139	140	**Sensitivity (%)**	**Specificity (%)**
**Total**		11	139	150	90.9	100.0
Day 9		**Real State**		Grayscale Values: 182.5		AUC: 0.99
		**TRUE**	**FALSE**	**Total**	**Accuracy (%)**	**Precision (%)**
**Predict**	**TRUE**	11	0	11	100.0	100.0
	**FALSE**	0	139	139	**Sensitivity (%)**	**Specificity (%)**
**Total**		11	139	150	100.0	100.0
Day 10		**Real State**		Grayscale Values: 181.0		AUC: 0.99
		**TRUE**	**FALSE**	**Total**	**Accuracy (%)**	**Precision (%)**
**Predict**	**TRUE**	11	0	11	100.0	100.0
	**FALSE**	0	139	139	**Sensitivity (%)**	**Specificity (%)**
**Total**		11	139	150	100.0	100.0

**Table 4:** Using detection device to identify and classify dead-in-shell eggs on the 7th–10th day.

Day 7		**Real State**		Grayscale Values: 122.0		AUC: 0.83
		**TRUE**	**FALSE**	**Total**	**Accuracy (%)**	**Precision (%)**
**Predict**	**TRUE**	4	8	12	92.1	33.3
	**FALSE**	3	124	127	**Sensitivity (%)**	**Specificity (%)**
**Total**		7	132	139	57.1	93.9
Day 8		**Real State**		Grayscale Values: 107.5		AUC: 0.95
		**TRUE**	**FALSE**	Total	Accuracy (%)	Precision (%)
**Predict**	**TRUE**	5	1	6	97.8	83.3
	**FALSE**	2	131	133	**Sensitivity (%)**	**Specificity (%)**
**Total**		7	132	139	71.4	99.2
Day 9		**Real State**		Grayscale Values: 74.0		AUC: 0.99
		**TRUE**	**FALSE**	**Total**	**Accuracy (%)**	**Precision (%)**
**Predict**	**TRUE**	7	0	7	100.0	100.0
	**FALSE**	0	132	132	**Sensitivity (%)**	**Specificity (%)**
**Total**		7	132	139	100.0	100.0
Day 10		**Real State**		Grayscale Values: 65.0		AUC: 0.99
		**TRUE**	**FALSE**	**Total**	**Accuracy (%)**	**Precision (%)**
**Predict**	**TRUE**	6	0	6	99.3	100.0
	**FALSE**	1	132	133	**Sensitivity (%)**	**Specificity (%)**
**Total**		7	132	139	85.7	100.0
